# Supplementary material for: Identification of photoperiod-induced specific miRNAs in the adrenal glands of Sunite sheep (Ovis aries)
Source: Front Vet Sci. 2022 Jul 22;9:888207. doi: 10.3389/fvets.2022.888207 (PMC9354845; doi:10.3389/fvets.2022.888207)
Supplement: Supplementary file 1 [file Data_Sheet_1.ZIP › Supplementary file/miRDeep2 file.zip!/miRDeep2 file/Novel_Structure/SP42A3/Novel_621.pdf]

Provisional ID	:	NC_019471.2_21010
Score total	:	72.5
Score for star read(s)	:	3.9
Score for read counts	:	64.4
Score for mfe	:	2.6
Score for randfold	:	1.6
Score for cons. seed	:	
Total read count	:	138
Mature read count	:	133
Loop read count	:	0
Star read count	:	5

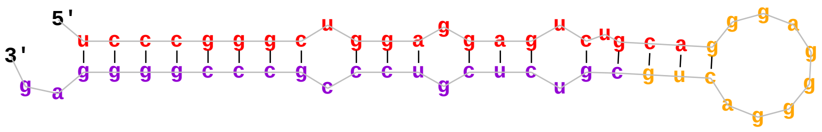

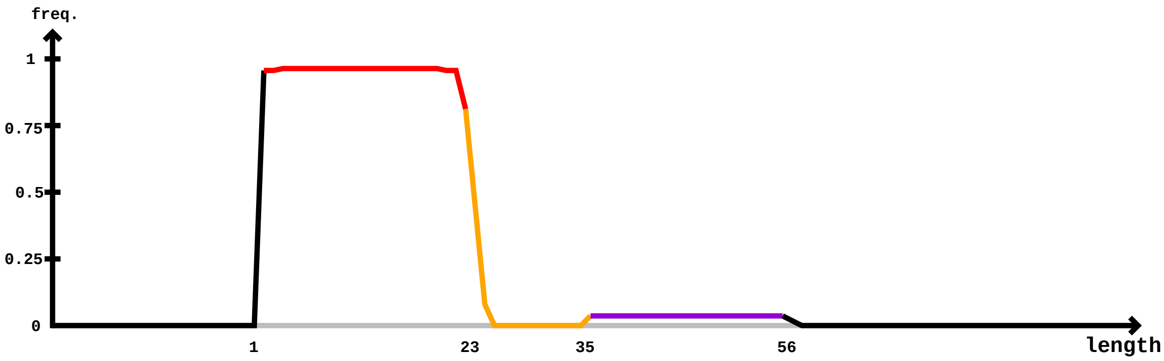

Mature Star

```
5'- ggaccugggcaggggcggggucccgggcuggaggagucugcagggaggggacugcgucucgucccgcccggggagaccgccccuugccgcucccaggacgguggauagcgg   -3'    obs
    ggaccugggcaggggcggggucccgggcuggaggagucugcagggaggggacugcgucucgucccgcccggggagaccgccccuugccgcucccaggacgguggauagcgg          exp
    .......((((((((((..((((((((.(((.(((.(.((((........))))).))).))).))))))))...))))))).))))(((.(((......)))..)))..   reads     mm     sample
    ....................ucccgggcuggaggagucu........................................................................   1         0      seq
    ....................ucccgggcuggaggagucugc......................................................................   20        0      seq
    ....................ucccgggcuggaggagucugca.....................................................................   64        0      seq
    ....................ucccgggcuggaggagucugcag....................................................................   36        0      seq
    ....................ucccgggcuggaggagucugcagg...................................................................   11        0      seq
    ......................ccgggcuggaggagucugca.....................................................................   1         0      seq
    ......................................................cgucucgucccgcccggggag....................................   4         0      seq
    ......................................................cgucucgucccgcccggggaga...................................   1         0      seq
```
